# Assessing urology and nephrology research activity in Arab countries using ISI web of science bibliometric database

**DOI:** 10.1186/1756-0500-7-258

**Published:** 2014-04-23

**Authors:** Waleed M Sweileh, Sa’ed H Zyoud, Samah W Al-Jabi, Ansam F Sawalha

**Affiliations:** 1 Department of Pharmacology and Toxicology, College of Medicine and Health Sciences, An-Najah National University, Nablus, Palestine; 2 Department of Clinical and Community Pharmacy, College of Medicine and Health Sciences, An-Najah National University, Nablus, Palestine

**Keywords:** Bibliometric, Urology and nephrology, Arab world, ISI Web of science

## Abstract

**Background:**

Bibliometric analysis is increasingly being used for research assessment. The main objective of this study was to assess research output in Urology and Nephrology subject from the Arab countries. Original scientific articles or reviews published from the 21 Arab countries in “Urology and Nephrology” subject were screened using the ISI Web of Science database. Research productivity was evaluated based on a methodology developed and used in other bibliometric studies by analyzing the annual productivity, names of journals, citations; top 10 active institution and authors as well as country contribution to Urology and Nephrology research.

**Results:**

Three thousand and seventy six documents in “urology and nephrology” subject category were retrieved from 104 journals. This represents 1.4% of the global research output in “urology and nephrology”. Four hundred and two documents (12.66%) were published in *Annales D Urologie Journal*. The *h*-index of the retrieved documents was 57. The total number of citations, at the time of data analysis, was 30401 with an average citation of 9.57 per document. Egypt, with a total publication of 1284 (40.43%) ranked first among the Arab countries in “urology and nephrology” subject category. Mansoura University in Egypt was the most productive institution with a total of 561 (15.33%) documents. Arab researchers collaborated most with researchers from the United States of America (226; 7.12%) in urology and nephrology research.

**Conclusion:**

The present data reveals a good contribution of some Arab countries to the field of “urology and nephrology”. More efforts are needed by some other Arab countries to bridge the gap in urology and nephrology research. Overall, the quality of urology/nephrology research is considered relatively high as measured by *h-*index. Cooperation in urology/nephrology research should be encouraged in the Arab world to bridge the gap with that from developed countries.

## Background

Medical education and clinical practice in many Arab countries have witnessed a dramatic change in the past three decades. Many medical schools, hospitals, and specialized medical research centers have been established. Research in the medical field reflects excellence and quality of medical education and clinical practice. Actually, quality and quantity of research output in any health subject reflects country’s interest and efforts to provide better health standards to the people of that country. One method to assess research output from any country is Bibliometric analysis which refers to the implementation of statistical methods for evaluating the research productivity, for individuals, institutes and countries [1]. Bibliometric analysis is a useful tool to obtain information about the current state of research in particular areas and allows researchers to identify and undertake new lines of research [2]. Bibliometrics has been applied to various diseases and is now widely accepted as a method of measuring research and literacy output in any particular area [3-8].

It is believed that medical research output from Arab countries is still lagging behind compared to non-Arab countries in the region like Israel, Turkey or Iran [9-14]. However, Egypt has surpassed Israel in Urology research but lags behind Israel in cardiology research [15]. Historically speaking, Arabs have made valuable contribution to medicine and urology [16]. Actually, pharmacological and surgical aspects of urology has been known in ancient Egypt [17]. Urology and nephrology are subjects of great importance in the Arab world since risk factors for such diseases, like diabetes mellitus, hypertension and obesity are prevalent in the Arab world. According to International Diabetes Federation, 6 out of the world’s top ten countries for highest prevalence (%) of diabetes are in the Middle East and North Africa Region–Kuwait, Lebanon, Qatar, Saudi Arabia, Bahrain and the United Arab Emirates [18]. Prevalence of hypertension, another important risk factor for chronic kidney disease (CKD), is also believed to be high among Arabs [19-21]. A recent mini review indicated that there is an urgent need for epidemiological studies about CKD in the Arab countries [22]. Arab researchers have established several peer reviewed journals dedicated for Urology and nephrology to encourage Arab researchers in this field [23].

The objective of this study was to analyze research output from 21 Arab countries in Urology and Nephrology. The Arab countries cover a large geographic area including North Africa and the Arabic Peninsula with around 500 million inhabitants. Studies regarding Urology and Nephrology research output have been published from several areas in the world [23-25]. However, up to the authors’ knowledge, no reports have been published from the Arab world about bibliometrics of research activity in “Urology and Nephrology” from the Arab world. Such bibliometric study will lead to a better understanding of the current and future status of Urology and Nephrology research in the Arab world which, hopefully, can lead to better preventive disease strategies and better patient-oriented health services [26-31]. The results of the study will help health policy makers and people in academia and clinical practice to shape up Urology and Nephrology research in the future. In addition, the momentum of research activity needs to be maintained through continuous analysis of publications from researchers in the region to provide feedback to academics, health institutions, and education planners.

## Methods

The data used in this study were based on the ISI Web of Science, which is one of the world largest databases of peer-reviewed literature. The world-leading citation databases provide authoritative, multidisciplinary coverage from more than 12,000 high impact research journals worldwide [32]. All Arab countries: Kingdom of Saudi Arabia (KSA); Egypt; Jordan; Lebanon; Qatar; Bahrain; Kuwait; Morocco; Tunisia; Syrian Arab Republic (SAR); United Arab Emirates (UAE); Iraq; Sudan; Yemen; Algeria; Comoros; Djibouti; Libya; Mauritania; Oman; Somalia, except Palestine, were used as country keys followed by “Urology and Nephrology” phrase as Web of Science Category. Palestine was excluded from search keys because the Web of Science database does not recognize Palestine as an independent state yet. The search keys looked like this: (CU = (Jordan) OR CU = (Iraq) OR CU = (Syria) OR CU = (Saudi) OR CU = (Kuwait) OR CU = (Egypt) OR CU = (Yemen) OR CU = (Qatar) OR CU = (Emirates) OR CU = (Bahrain) OR CU = (Oman) OR CU = (Sudan) OR CU = (Tunisia) OR CU = (Algeria) OR CU = (Lebanon) OR CU = (Libya) OR CU = (Morocco) OR CU = (Somalia) OR CU = (Djibouti) OR CU = (Comoros) OR CU = (Mauritania)) AND WC = (Urology and Nephrology). To increase the accuracy of results, research was refined and limited to original research articles and review articles because they represent the research activities, while other types of documents like editorials, conference proceedings, and others were excluded. The time frame for the result was up to year 2011. The 2012 and 2013 years were excluded because they are still open of new journal issues.

The database then generates a count of the total number of original articles, the total citations, and the value of the *h*-index (highly cited index). The *h*-index represents the number of citations received for each of the documents in descending order, while the h-graph measures the impact of a set of documents and displays the number of citations per document (for example: h-index of 10 means that there are 10 items that have 10 citations or more). The h-index was originally developed as a measure of qualifying research performance [33,34]. The collected data were used to generate the following information: (a) total and trends of contributions in Urology and Nephrology research during all previous years up to the set date of data analysis (December 31th, 2011); (b) Arab countries research productivity and collaboration patterns; (c) journals in which Arab world researchers published; and (d) the citations received by the publications.

### Ethical approval

The Institutional Review Board (IRB) at An-Najah National University does not require submission of an IRB application for such study. The IRB considered that there is no risk for human subjects in such publications since the data are based on published literature and did not involve any interactions with human subjects.

### Statistical analysis

Data from ISI Web of Science were exported to Microsoft Office Excel® and then transferred to the Microsoft word program. The measurements of bibliometric analysis (e.g. countries, cited articles, institutions) were converted to the rank order using the standard competition ranking (SCR). We took into consideration the top 10 ranking in each item. If the measurements of bibliometric analysis have the same ranking number, then a gap is left in the following ranking numbers. The journal’s impact factors (IF) were evaluated using the Journal Citation Report (JCR; Web of Knowledge) 2012 science edition by Thomson Reuters (New York, NY, USA).

## Results

**Figure 1:**
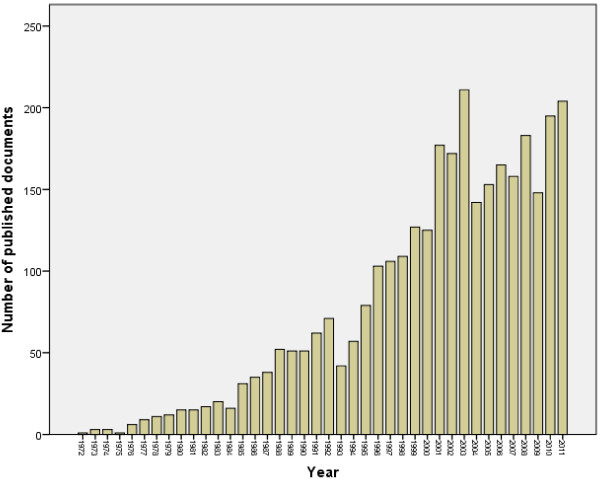
Growth of urology/nephrology research in Arab countries as extracted from ISA web of science using urology and nephrology as Web of Science Category.

**Figure 2:**
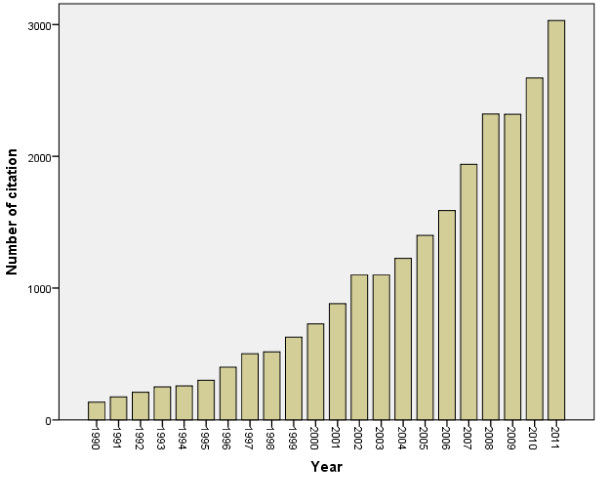
Changes in total number of citation in the past 2 decades for urology & nephrology documents published from Arab countries.

The total number of documents retrieved from ISI Web of Science using “Urology and Nephrology” subject category without specifying the name of any country was 224,479. This number represents the global research productivity (original research articles and reviews) in urology and nephrology subject up to year 2011. When the same methodology was applied using the list of the 21 Arab countries, 3176 documents were retrieved. Therefore, research output in urology and nephrology from Arab countries represents 1.4% of the global research productivity in urology and nephrology. The 3176 consisted of 3049 (96.0%) original journal articles, and 127 (4%) review articles. The annual number of documents published from Arab countries indicated that urology and nephrology research output remained low until mid-1990s (Figure 1). More than 50% of documents were published after the year 2001. The language of most documents was English (2366; 74.5%) followed by French (788; 24.81%), Dutch (14; 0.44%) and Spanish (8; 0.25%). The first urology and nephrology article from Arab countries was published in 1972 in *Annales d’Urologie* by Dr. Said, H from Tunisia and the title of the article was “Pelvic and lower lumbar ectopy of kidney in children-with 7 case reports” [35]. Figure 2 shows the changes in the total number of citations in each year which reflects the changes in quality of publication in urology & nephrology from Arab countries.

The retrieved documents were published in 104 peer-reviewed urology and nephrology journals registered in Web of Knowledge®. Four hundred and two articles (12.66%) were published in *Annales D Urologie Journal* which started in 1969 and was discontinued as of 2007. Table 1 lists the top 10 journals in which documents in urology and nephrology were published from the Arab countries. Of the 3176 urology and nephrology documents, there were 131 documents in the transplantation, 111 in the pediatric and 47 in the obstetric/gynecology research area. Other overlapping research areas of documents in urology and nephrology field are shown in Table 2.

**Table 1:** Top 10 journals in which urology and nephrology documents from the 21 Arab countries were published

**SCR**^**a**^	**Journal name**	**Number of documents**	**IF**^**b**^
		**N (100%) = 3176**	
**1**^**st**^	*Annales D Urologie*	402 (12.66)	NA^c^
**2**^**nd**^	*Journal of Urology*	313 (9.86)	3.696
**3**^**rd**^	*BJU International (British Journal of Urology)*^d^	307 (9.67)	3.046
**4**^**th**^	*Progres en Urologie*	225 (7.08)	0.801
**5**^**th**^	*Urology*	207 (6.52)	2.424
**6**^**th**^	*Journal of Sexual Medicine*	97 (3.05)	3.513
**6**^**th**^	*Nephrology Dialysis Transplantation*	97 (3.05)	3.371
**6**^**th**^	*Scandinavian Journal of Urology and Nephrology*	97 (3.05)	1.007
**9**^**th**^	*Journal D Urologie*	94 (2.96)	2.708
	*Changed to Archives of Dermatological Research*		
**10**^**th**^	*Journal of Endourology*	89 (2.80)	2.074

*Abbreviations*: *SCR* standard competition ranking; *NA* not available; *IF* impact factor.

^a^Equal journals have the same ranking number, and then a gap is left in the ranking numbers.

^b^The impact factor was reported according to Institute for Scientific Information (ISI) journal citation reports (JCR) 2012.

^c^Discontinued on 2007.

^d^Started on 1929 as British Journal of Urology and was discontinued on 1998. Continued as BJU International.

**Table 2:** Research categories (WOS categorization) of the 3176 urology nephrology documents published from the 21 Arab countries

**Research area**	**Number of documents**
	**N (100) = 3176 (100%)**
Urology and nephrology	3176 (100)
Transplantation	131 (4.13)
Pediatrics	111 (3.50)
Obstetrics gynecology	47 (1.48)
Engineering	34 (1.07)
Endocrinology metabolism	31 (0.98)
Oncology	28 (0.88)
Physiology	20 (0.63)
Cardiovascular system cardiology	15 (0.47)
Hematology	15 (0.47)
Public environmental occupational health	10 (0.32)
Nutrition dietetics	7 (0.22)
Research experimental medicine	4 (0.13)
Gastroenterology hepatology	2 (0.06)
Cell biology	1 (0.03)
General internal medicine	1 (0.03)
Materials science	1 (0.03)
Pharmacology pharmacy	1 (0.03)
Surgery	1 (0.03)

When retrieved data were analyzed by country (Table 3), Egypt (1248; 40.43%) had the highest research output followed by Morocco (553; 17.41%) and Kingdom of Saudi Arabia (513; 16.15%). No data related to urology and nephrology was found from Djibouti, Mauritania and Comoros. Researchers from other countries have collaborated with researchers in the Arab world in Urology and Nephrology. Countries whose researchers collaborated most with researchers in the Arab world include the United States of America (USA); (226; 7.12%) followed by France (102; 3.21%) and Canada (98; 3.09%) (Table 4). The total number of citations, at the time of data analysis (February 16^th^, 2014), was 30401 with an average citation of 9.57 per document. Of the 3176 documents considered for the *h*-index, 57 had been cited at least 57 times at the time of data analysis.

**Table 3:** Contribution of each Arab country to the 3176 documents published in the subject “urology and nephrology”

**Country**	**Number of documents**
	N (%) = 3176 (100%)*
Egypt	1284 (40.43)
Morocco	553 (17.41)
Kingdom of Saudi Arabia	513 (16.15)
Tunisia	374 (11.78)
Kuwait	156 (4.91)
United Arab Emirates	98 (3.09)
Lebanon	76 (2.39)
Jordan	65 (2.05)
Qatar	35 (1.10)
Sudan	33 (1.04)
Iraq	27 (0.85)
Oman	26 (0.82)
Algeria	25 (0.79)
Libya	13 (0.41)
Yemen	10 (0.28)
Bahrain	7 (0.22)
Somalia	4 (0.13)
Syria	4 (0.13)
Comoros	0 (0.0)
Djibouti	0 (0.0)
Mauritania	0 (0.0)

*total exceeds 100% because of overlap in some documents among more than one Arab country.

**Table 4:** Top 10 countries whose researchers have collaborated with Arab researchers in publication of the 3176 documents in urology and nephrology

**SCR**	**Country**	**Number of documents**
		**N (%) = 3176 (100%)**
1^st^	United States of America	226 (7.12)
2^nd^	France	102 (3.21)
3^rd^	Canada	98 (3.09)
4^th^	England	84 (2.65)
5^th^	Germany	77 (2.42)
6^th^	Netherlands	39 (1.23)
7^th^	Sweden	34 (1.07)
8^th^	Japan	27 (0.85)
9^th^	Belgium	24 (0.76)
10^th^	Italy	23 (0.72)

Table 5 shows the top 10 most productive Arabic institutions in urology and nephrology field. The most productive institution was Mansoura University (561; 17.71%). Table 6 presents the top 10 ranking of prolific authors who published in urology and nephrology field from the Arab world with their affiliations. Table 7 presents a list of the 10 most cited urology and nephrology articles originated from Arab countries [36-45]. It is noteworthy that 9 out of the top 10 cited articles from Arab countries were in clinical urology/nephrology rather than basic science of urology/nephrology.

**Table 5:** Top 10 active institutions in the field of urology and nephrology in the 21 Arab countries

**SCR**^**a**^	**Institute**	**Country**	**Number of documents**
**1**^**st**^	Mansoura University^b^	Egypt	561 (15.33)
**2**^**nd**^	Cairo University	Egypt	323 (10.17)
**3**^**rd**^	CHU Ibn Sina^c^	Morocco	153 (2.46)
**4**^**th**^	Kuwait University	Kuwait	123 (3.87)
**5**^**th**^	King Saud University	KSA	116 (3.65)
**6**^**th**^	King Faisal Specialist Hospital Research Center	KSA	112 (3.53)
**6**^**th**^	CHU Ibnou Rochd^d^	Morocco	136 (3.31)
**6**^**th**^	HOP Charles Nicolle	Tunisia	103 (3.24)
**9**^**th**^	Alexandria University	Egypt	95 (2.99)
**10**^**th**^	Ain Shams University	Egypt	83 (2.61)

*Abbreviations*: *SCR* standard competition ranking; *KSA* Kingdom of Saudi Arabia.

^a^Equal institutions have the same ranking number, and then a gap is left in the ranking numbers.

^b^represents productivity from Mansoura University and Urology Nephrology Center/Mansoura.

^c^represents CHU Ibn Sina, CHU AVICENNE and HOP AVICENNE.

^d^represents CHU Ibnou Rochd and CHU Ibn rochd.

**Table 6:** Ranking of the top 10 prolific authors who published in the field of urology and nephrology from the 21 Arab countries or who collaborated with authors located in the Arab countries

**SCR**^**a**^	**Author**	**Number of published documents**	**Affiliation as shown in ISI web of knowledge last publication**
		**N (%) = 3176 (100%)**	
**1**^**st**^	Ghoneim, M.A	165 (5.2)	Mansoura Univ, Dept Urol, Urol & Nephrol Ctr, Mansoura, Egypt
**2**^**nd**^	Shokeir, A.A	116 (3.65)	Mansoura Univ, Urol & Nephrol Ctr, Dept Urol, Mansoura, Egypt
**3**^**rd**^	Benchekroun, A	107 (3.37)	CHU Ibn Sina, Serv Urol A, Rabat, Morocco
**4**^**th**^	Benjelloun, S	104 (3.28)	Ctr Hosp Ibn Rochd, Serv Urol, 25, Rue Rome,Angle Rue Amsterdam, Casablanca, Morocco.
**5**^**th**^	Shafik, A	88 (2.77)	Cairo Univ, Dept Surg & Expt Res, Fac Med, Cairo 11121, Egypt
**6**^**th**^	Faik, M	85 (2.68)	CHU Rabat, Hop Ibn Sina, Serv Urol A, Rabat, Morocco
**7**^**th**^	Lakrissa, A	74 (2.33)	Ibn Sina Hosp, Dept Urol B, Univ Teaching Ctr, Rabat, Morocco
**8**^**th**^	Hachimi, M	72 (2.27)	CHU Ibn Sina, Serv Urol, Rabat, Morocco
**8**^**th**^	Joula, A	72 (2.27)	CHU Ibnou Rochd, Serv Urol, Casablanca, Morocco
**10**^**th**^	Ayed, M	68 (2.14)	Hop Charles Nicolle, Dept Urol, Tunis, Tunisia

*Abbreviation*: *SCR* standard competition ranking.

^a^Equal authors have the same ranking number, and then a gap is left in the ranking numbers.

^b^Percentage of publications for each author by the total number of documents.

**Table 7:** Top 10 cited articles in the subject “urology and nephrology” that were authored/ co-authored by researchers from 21 Arab countries

**SCR**	**Article title**	**Name of journal**	**Year; authors as shown in ISI**	**Type of study (Basic versus Clinical)**	**Number cited**
1^st^	Laparoscopic versus open radical nephrectomy: A 9-year experience	Journal of Urology	2000; Dunn, MD; Portis, AJ; Shalhav, AL; et al. [36]	Clinical	319
2^nd^	Worldwide ethnic distribution of the G protein beta 3 subunit 825 T allele and its association with obesity in Caucasian, Chinese, and Black African individuals	Journal of the American Society of Nephrology	1999; Siffert, W; Forster, P; Jockel, KH; et al. [37]	Basic	265
3^rd^	Radical cystectomy for carcinoma of the bladder: Critical evaluation of the results in 1,026 cases	Journal of Urology	1997; Ghoneim, MA; ElMekresh, MM; ElBaz, MA; et al. [38]	Clinical	240
4^th^	An International Urogynecological Association (IUGA)/International Continence Society (ICS) joint report on the terminology for female pelvic floor dysfunction	International Urogynecology Journal	2010; Haylen, Bernard T.; de Ridder, Dirk; Freeman, Robert M.; et al. [39]	Clinical	218
5^th^	Vascular access for hemodialysis	Kidney International	1999; Schwab, SJ; Harrington, JT; King, AJ; et al. [40]	Clinical	204
6^th^	Nerve-sparing robot-assisted radical cystoprostatectomy and urinary diversion	BJU International	2003; Menon, M; Hemal, AK; Tewari, A; et al. [41]	Clinical	199
7^th^	Extended radical lymphadenectomy in patients with urothelial bladder cancer: Results of a prospective multicenter study	Journal of Urology	2004; Leissner, J; Ghoneim, MA; Abol-Enein, H; et al. [42]	Clinical	193
8^th^	Causes, kinetics and clinical implications of post-hemodialysis urea rebound	Kidney International	1988; Pedrini, LA; Zereik, S; Rasmy, S. [43]	Clinical	174
9^th^	The burden of kidney disease: Improving global outcomes	Kidney International	2004; Eknoyan, G; Lameire, N; Barsoum, R; et al. [44]	Clinical	144
10^th^	Laparoscopic nephroureterectomy for upper tract transitional cell cancer: The Washington University experience	Journal of Urology	2000; Shalhav, AL; Dunn, MD; Portis, AJ; et al. [45]	Clinical	142

## Discussion

In the present study, bibliometric analysis of Urology and Nephrology research activity from 21 Arab countries was carried out. Our study analyzed a total of 3167 documents extracted from ISI Web of Science using Subject key (Urology and Nephrology) and using country affiliation of the 21 Arab countries. Therefore, our results do not include Urology and Nephrology literature outside ISI Web of Science database or literature published in journals not categorized in the field of urology and nephrology. For example, articles pertaining to urology and nephrology that were published in *Arab Journal of Urology* and in *Saudi Journal of Kidney Diseases and Transplantation* were not included in the analysis since these journals are not indexed in ISI Web of Science. Despite that, our study does give a clear picture about the characteristics of research from Arab countries published in international journals, especially those indexed by Web of Science under the category (Urology and Nephrology”). Although bibliometric analysis might slightly differ from one search engine to another, Web of Science search engine remains one of the best available tools for analyzing and tracking citations [46,47]. A study that compared PubMed, Scopus, Web of Knowledge, and Google Scholar has found that PubMed remains an important resource for clinicians and researchers, while Web of Knowledge covers a wider journal range and offers the capability for citation analysis [46-49].

The data obtained in our study will serve as a baseline data for evaluation of future research activities and for comparative purposes with other non-Arab countries. A study carried out to perform a bibliometric evaluation of publications from European Union (EU) countries in the international urological journals between 2000-2005 has indicated that such studies demonstrated a feasible solution to validate and compare the contribution of the various EU countries towards the urological research [50]. It is believed that basic and clinical research in urology and nephrology promotes clinical practice. For example, clinical research have promoted the development of nephrology in China [51].

The annual number of documents published under Urology and Nephrology category from the 21 Arab countries was acceptable although the general medical research activity from Arab countries is low [13,52-55]. Studies suggested that the lack of funding, freedom, and democracy may contribute to low scientific research output in the Arab world [13,52,53,55]. Our study showed that there were some countries, such as, Egypt and Morocco, KSA where their total research productivity was clearly higher than that in the remaining countries. Previous studies reported that Egypt and KSA had high biomedical and nephrology publications among the Arab countries [13,56,57], which is consistent with the current results in the field of urology and nephrology research publications.

Our results showed that authors from Arab region mainly collaborated with authors from USA, France and Canada. This may be because most academics from Arab countries graduated from or were trained in these countries. International collaboration is beveled to increase the quantity and quality of research productivity [58,59]. Research collaboration is an important mechanism to improve quality and quantity of research at the university level. A study has found that there is a positive correlation between research productivity, funding, public impact and international and domestic collaboration at the author level [60-62]. A study has found that at article level, both within-university collaboration and international collaboration are positively related to an article’s quality and that, at scientist-year level, only international collaboration is positively related to a scientist’s future research output [63]. Other studies indicated that international collaboration can increase the visibility of scientific publication from a particular country [64]. Furthermore, international collaboration in research helps in capacity building in developing countries and make national problems of developing countries more observable [65].

Research output studies in the field of Urology and Nephrology have been conducted in several parts of the world [23-25,50,66-69]. A recent Iranian study using Medline database and the IranMedex between 1997 and 2007 reported that (1) the total number of publications in the field kidney disease from Iran was 579 (average of 58 papers per year); (2) more than 56% of the publications (324) were in journals that were indexed in the Medline; and (3) the majority of the papers were concerned with transplantation (58.3%), nephrology (20.0%), and hemodialysis (16.8%). Authors of the Iranian study concluded that Iran’s contribution to the research on kidney disease is not satisfactory in terms of the volume and quality of publications. On the other hand, the data suggest that there was a significant research activity in the field of kidney transplantation during the studied period [24]. A Japanese study indicated that Japan’s share of research output for selected journals in urology and nephrology was 6% and it ranked second in the world following the USA [66]. A study analyzed the characteristics of highly cited articles showed that highly cited articles are very different from ‘ordinary’ cited articles and that highly cited articles are usually authored by a large number of scientists and often involving international collaboration [70]. Highly cited articles positively contribute to the *h*-index of the individual author and to the institution and country [71-74]. The citation is a key indicator of research quality and researchers need to be aware of mechanisms that might enhance citation of published articles like self-citation whenever possible [75].

To the best of the authors’ knowledge, our study is the first article to analyze the quantity and quality of research productivity in the field of urology and nephrology from the Arab world. Our study showed that some Arab countries, such as Egypt and KSA, clearly had higher research urology and nephrology research output than the remaining Arab countries. This high activity is due to population, national income and overall scientific activity of these countries. The main goal of this paper was to direct attention and to open the doors for a scientific discussion among professionals and academics. It is recommended that similar quantitative and qualitative research analyses for other disciplines, based on the same methodology, should be made for Arab countries. This would provide a more comprehensive picture of the overall research productivity both at the regional level and at the international level.

Finally, our study is not without limitations, most of which are the same as those of studies performed in other biomedical fields [7,8,14,76]. First of all, we used ISI web of knowledge database and therefore articles published in elsewhere were not included. Second, many articles in urology and nephrology might have been published in non-urology/non-nephrology journals.

## Conclusion

The present data show promising and relatively good urology/nephrology research productivity from Arab countries especially in the last decade. However, wide variation in research productivity among Arab countries in urology/nephrology do exists. Egypt is the leading country in this regard and Mansoura University is leading institution in Arab country in the field of urology/nephrology research. The quality of urology/nephrology research from Arab countries is also good and promising. The methodology used in this manuscript can be applied to other fields of medical science for comparison of research activity in the Arab world with that in non-Arab countries. Cooperation in urology/nephrology research should be encouraged in the Arab world to bridge the gap with other developed countries.

## Abbreviations

CKD: Chronic kidney disease; SPSS: Statistical package for social sciences; ISI: Institute for Scientific Information; KSA: Kingdom of Saudi Arabia; UAE: United Arab Emirates; SAR: Syrian Arab Republic; USA: United States of America; EU: European Union; JCR: Journal citation report; IRB: Institutional Review Board; SCR: Standard competition ranking; IFs: Impact factors.

## Competing interests

The authors declare that they have no competing interests.

## Authors’ contributions

All authors were involved in drafting the article and all authors approved the final version to be submitted for publication. All authors have added an intellectual significant value to the manuscript. WS and SZ were involved in study design and concept. SA and AS were involved in data analysis and manuscript writing. All authors read and approved the final manuscript.
